# Duck cGAS inhibits DNA and RNA virus replication by activating IFNs and antiviral ISGs

**DOI:** 10.3389/fimmu.2023.1101335

**Published:** 2023-01-17

**Authors:** Chang Lin, Min Zheng, Shifeng Xiao, Shao Wang, Xiaoli Zhu, Xiuqin Chen, Dandan Jiang, Xiancheng Zeng, Shaoying Chen, Shilong Chen

**Affiliations:** ^1^ College of Animal Sciences, Fujian Agriculture and Forestry University, Fuzhou, Fujian, China; ^2^ Laboratory of Animal Virology, Institute of Animal Husbandry and Veterinary Medicine, Fujian Academy of Agriculture Sciences, Fuzhou, Fujian, China; ^3^ College of Life Sciences, Longyan University, Longyan, China

**Keywords:** duck, innate immunity, cyclic GMP-AMP synthase, antiviral function, interferon-stimulated genes, sgRNA

## Abstract

Cyclic GMP-AMP Synthase (cGAS) is a pivotal adaptor of the signaling pathways involving the pattern recognition receptors and plays an important role in apoptosis and immune regulation. The cGAS function in mammals has been investigated extensively; however, the function of duck cGAS (du-cGAS) in response to viral infections is still unclear. This study aimed to clone the mallard (*Anas platyrhynchos*) cGAS homolog to investigate the function of duck cGAS (du-cGAS) in host antiviral innate immunity. The results showed that the open reading frame (ORF) region of the du-cGAS gene was 1296 bp, encoding 432 amino acids (aa) and exhibiting similar functional domains with its chicken counterpart. Knockdown of the endogenous du-cGAS by specific sgRNA strongly increased the replication of DNA viruses, including duck adenovirus B2 (DAdV B2) and duck short beak and dwarfism syndrome virus (SBDSV). However, the knockout did not impair the replication of novel duck reovirus (NDRV), an RNA virus. Furthermore, the mRNA expressions of type I interferon (IFNs) and vital interferon-stimulated genes (ISGs) were remarkably reduced in the du-cGAS knockout DEF cell line. Inversely, du-cGAS overexpression greatly activated the transcription of IFN-α, IFN-β, and vital ISGs, and impaired the replication of DAdV B2, SBDSV, and NDRV in the DEF cell line. Importantly, we found that a deletion of 68 aa in the N terminus didn’t impair the antiviral function of du-cGAS. Overexpressing NTase Core, C-Domain (Mab21), or Zinc-Ribbon domain independently had no antiviral effects. Generally, these results reveal that du-cGAS is a vital component of the innate immune system of ducks, with a universal antiviral activity, and provides a useful strategy for the control of waterfowl viral diseases.

## Introduction

The host innate immune system is the first line of defense against viral invasion (1). The innate immune system recognizes the recognition of pathogen-associated molecular patterns (PAMPs) of the invading viruses *via* the pattern recognition receptors (PRRs) (2). The PRRs activated upon PAMPs recognition interact with their downstream signaling proteins to activate and upregulate innate immune transcription factors such as NF- κB and the interferon regulatory factor-3 and factor-7 (IRF3 and IRF7) (2). This induces the translocation of regulatory factors to the nucleus, which upregulates the expression of type I and III interferons (IFNs) (3). IFNs activate the JAK-STAT pathway and promote the expression of various IFN-stimulated gene (ISG) family proteins (4). These ISG proteins are important effector molecules with varying antiviral effects capable of blocking the different life stages of the virus, including viral entry, translation, replication, assembly, and spread (5, 6).

Cyclic GMP-AMP synthase (cGAS), a cytoplasmic DNA sensor belonging to the N-tases family, consists of three domains: an N-terminal helix extension domain, a conserved NTase domain, and a C-terminal domain (Mab21) (7). cGAS exists as a monomer in the resting state. However, upon recognizing and binding intracellular pathogenic DNA, such as bacteria, DNA viruses, and retroviruses, cGAS forms a 2:2 complex in the cytoplasm (8). This complex induces a conformational change in the cGAS active site, which catalyzes the synthesis of the second messenger cGAMP from ATP and GTP (9). cGAMP binds to the stimulator of interferon genes (STING) localized at the endoplasmic reticulum (ER) membrane (10). Upon activation, STING, an important signaling component of the DNA sensing pathway that induces type-I IFN production, recruits TANK-binding kinase-1 (TBK1) to form a STING-TBK1 complex (9, 11). The STING-TBK1 complex then translocates from the ER to the perinuclear lysosomal compartment through an autophagy-like process and subsequently activates the transcription factors IRF3 and NF- κB (9, 10, 12).

The cGAS-STING signaling pathway plays a pivotal role in the host defense against viral infections, such as SARS-CoV-2 and Ectromelia Virus (13, 14). Studies have shown that cGAS or STING-deficient mice are more susceptible to lethal infection after exposure to various DNA viruses, including herpes simplex virus 1 and ectromelia virus (14, 15). Recently, the cGAS-STING pathway is also involved in resistance against some bacterial infections (16). Unlike bacteria, many viruses have developed complex strategies to inhibit or escape the innate immune responses by negatively regulating the cGAS-STING pathway (17–21).

Despite the involvement of cGAS in antiviral responses, little is known about its ability to restrict duck-origin virus infection. This study evaluated the effects of duck cGAS (du-cGAS) in response to viral infections. We cloned the du-cGAS and prepared the mouse anti-du-cGAS antibodies. The CRISPR/Cas9 mediated gene editing system was used to develop the du-cGAS knockout cell. Silencing of the endogenous du-cGAS downregulated type-I IFNs and the downstream ISGs, enhancing DAdV-B2 and SBDSV replication. Overexpressing du-cGAS significantly increased the expressions of STING, type-I IFNs, and ISGs, and significantly inhibited the replication of DAdV-B2, SBDSV M15, and NDRV. These results revealed that du-cGAS is a critical component of the innate immune system of ducks, which inhibits virus infection.

## Materials and methods

### Cells and virus

The duck embryo fibroblast (DEF) cell line was obtained from the American type culture collection (ATCC) and cultured in Dulbecco’s modified Eagle medium (DMEM, Corning, China) containing 10% fetal bovine serum (FBS) (Sigma, US), followed by incubation in 5% CO_2_ at 37°C. Three virus strains, NDRV NP03, DAdV-B2 BG61, and SBDSV M15, previously preserved in our laboratory, were propagated in DEFs, and the virus titers were determined by median tissue culture infective dose (*TCID*_50_) assay using the Reed and Muench calculation.

### Du-cGAS cloning, prokaryotic expression, and anti-duck cGAS antibodies preparation

Mab21, 122aa-423aa domain of du-cGAS, was cloned into pET28a(+) using specific primers shown in **Table 1** to create a du-Mab21^-^pET28a(+) recombinant plasmid. The recombinant plasmid was then transformed into *E. coli BL21* for expressing the recombinant protein cGAS-Mab21 using a previously described method (22). The Balb/c mice were immunized with recombinant protein at an immunization interval of two weeks. Anti-Mab21 sera of du-cGAS protein was collected on day 7 after four immunizations.

**Table 1:** Primer sequences used in this study.

Primer name	Primer sequence (5′-3′)
pdu-cGAS-F	TTTAGTGACCGTCAGAATTCatggagggccccgggga
pdu-cGAS-R	TCCTTGTAATCGGTACCGGATCCatatccctgctgaaatattg
pdu-NTase-F	TTTAGTGACCGTCAGAATTC atgagggacggcggcttcgg
pdu-NTase-R	TCCTTGTAATCGGTACCGGATCCaacttccaaagccaagatg
pdu-Mab21-F	TTTAGTGACCGTCAGAATTCatgagctactacgagcgcgtcaa
pdu-Mab21-R	TCCTTGTAATCGGTACCGGATCCatatccctgctgaaatattg
pdu-Zinc-F	TTTAGTGACCGTCAGAATTCatgcgaatctctttctcacatat
pdu-Zinc-R	TCCTTGTAATCGGTACCGGATCCatatccctgctgaaatattg
du-cGAS-sgRNA-F	CACCgtcggggctggtgaaccag
du-cGAS-sgRNA-R	Aaaccctggttcaccagccccgac
SBDSV-VP3-F	gaggtagacagcaacagaaa
SBDSV-VP3-R	gctcgtccgtgaccata
DAdV-B2-Hexon-F	gaggtagacagcaacagaaa
DAdV-B2-Hexon-R	gctcgtccgtgaccata
NDRV-σB-F	ttatcagggtcggcaacgc
NDRV-σB-R	tcctgaggtcgcttactcgc

Uppercase DNA sequences are from plasmid and lowercase DNA sequences are from du-cGAS.

### Generation of du-cGAS knockout cell line

The specific single-guide RNAs (sgRNAs) targeting the du-cGAS were designed using the CRISPR guide RNA designing website (http://crispr.mit.edu/). The designed sequences are shown in **Table 1**. The synthesized oligonucleotide pairs were annealed and cloned into pX459-v2 CRISPR/Cas9 to construct the recombinant plasmid, as previously described (23). To generate the cGAS-knockout DEF cells, we transfected Du-cGAS-px459 into ATCC DEF using PolyFast Transfection Reagent (MCG, US), according to the manufacturer’s instructions. After 48h of transfection, puromycin (0.5 μg/ml) was added to the culture solution to select the positive DEFs. Thereafter, du-cGAS-knockout was verified by western blotting and DNA sequencing. The Du-cGAS knockout cell line was infected with DAdV-B2, SBDSV, and NDRV at the multiplicity of infection (MOI) of 0.25. The treatment and control cells were collected at three time points (24, 48 and 72 hours post-infection, hpi) for total RNA and protein extraction. Moreover, viral replication and gene mRNA expressions were measured by western blotting and qRT-PCR, respectively, as described previously (24). Supernatants were collected to detect the viral titers using the median tissue culture infectious dose (TCID_50_) assay.

### Construction of truncated du-cGAS overexpression plasmids

Different functional domains of Du-cGAS, including cGAS, cGAS with 68 aa deletion at the N terminus (cGAS (69aa-423aa)), Mab21 (122aa-423aa), NTase Core (70aa-238aa), and Zinc-Ribbon (289aa-358aa), were cloned into pFlag-CMV5a vector to generate truncated du-cGAS proteins according to a previous method (24). The primers used for cloning are shown in **Table 1**. DEF cells with 60–80% confluence was transfected with 6 µg of plasmid DNA per well using PolyFast Transfection Reagent. After 24 hours post-transfection, the DEF cells were infected with DAdV-B2, SBDSV, and NDRV strains at the MOI of 1 and harvested at 12, 24 and 36 hpi for qRT-PCR and western blotting analysis. The culture supernatants were then used for viral load detection using TCID_50_ assay. The infected cells were collected for RNA and protein extraction. Viral replication was measured by qRT-PCR and western blotting analysis, as described previously (24).

### Immunofluorescence assay and western blotting analysis

Cell monolayers with or without DAdV B2 infection were subjected to indirect immunofluorescence assay (IFA) to detect the DAdV B2 hexon protein expression. After incubated with primary and secondary antibody as the method described previously (25, 26), the cells were subsequently treated with 4′,6-diamidino-2-phenylindole (DAPI) for 10 min. The fluorescent signals were captured using a BZ-X800 confocal microscope (KEYENCE, Japan). Cell lysates were separated by 12% SDS-PAGE gel (Yamei, China) and transferred to polyvinylidene difluoride (PVDF) membranes. The PVDF membranes were blocked using 3% bovine serum albumin in phosphate-buffered saline (BSA-PBST) at room temperature for 2 h. The membranes were then incubated overnight with indicated primary antibodies (including monoclonal antibodies against DAdV B2, NDRV, SBDSV, and Flag-tag and the mouse anti-du-cGAS sera) at 4°Cand washed with Tris-buffered saline (TBS), followed by incubation with Daylight 680 Affineur Goat Anti-Mouse IgG (1:20,000) (Odyssey, US) at room temperature for 1h. Thereafter, the membranes were imaged using the Odyssey^®^ DLx (Bio-Techne, USA).

### qRT-PCR assay

Total RNA was extracted using the FastPure Cell/Tissue Total RNA Isolation Kit (Vazyme, Nanjing, China) containing genomic DNA Eraser, according to the manufacturer’s instructions. Purified RNA was reverse transcribed into cDNA using EasyScript First-Strand cDNA Synthesis SuperMix (Tranz, Beijing). Thereafter, qRT-PCR was performed using the PerfectStart Green qPCR SuperMix (Tranz, Beijing) on the Roche 96 Light Cycler (Roche, US), as previously described. The primer sequences used in this analysis have been described in a previous study (27). The primer sequences of NDRV σB and DAdV-B2 are shown in **Table 1**. The relative expression of each target gene was analyzed using the 2^-ΔΔct^ method, with duck GAPDH as a housekeeping gene.

### Statistical analyses

All data were presented as mean ± standard deviation (SD). The student’s t-test was used to determine the statistically significant differences using the Prism 8 software. A significance level of *P* < 0.05 was considered significant, *P* < 0.01 was considered highly significant, and *P* < 0.001 was extremely significant.

## Results

### Molecular characteristics of du-cGAS

**Figure 1:**
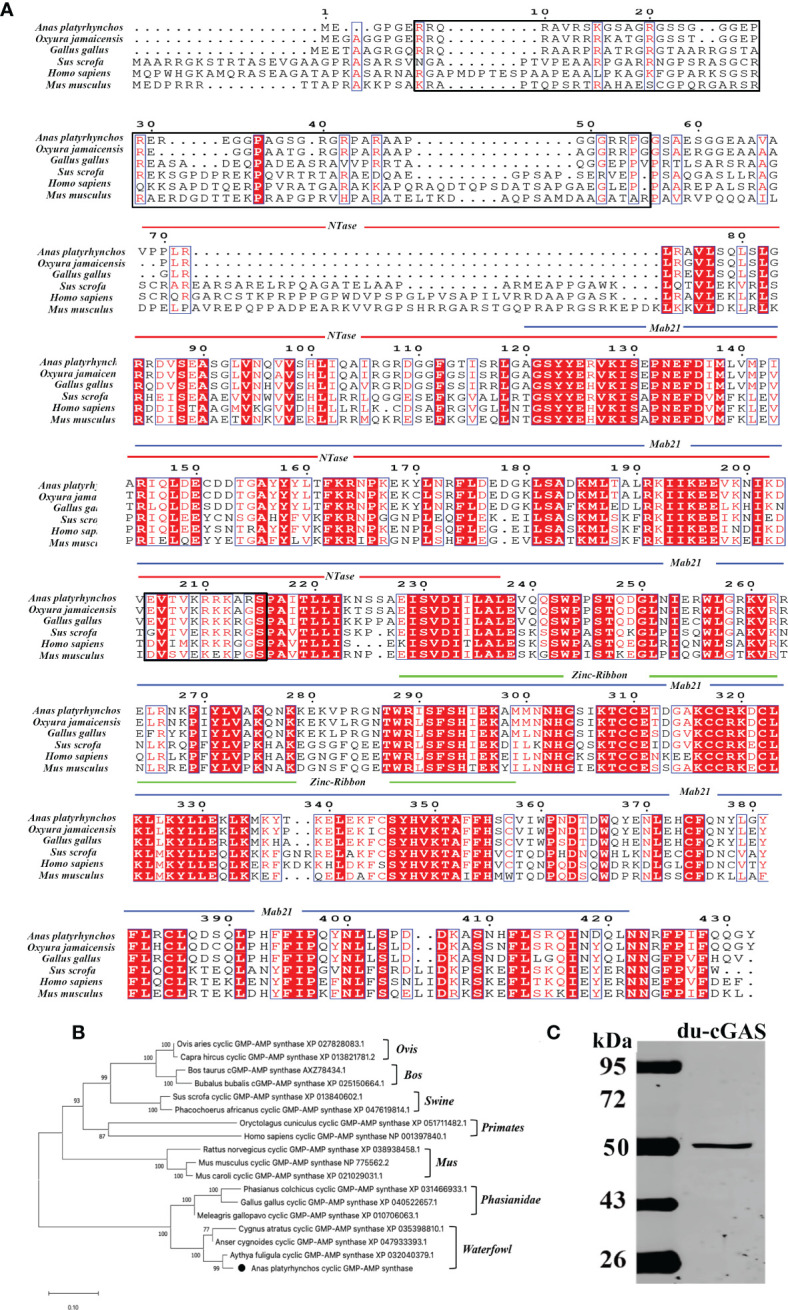
Amino acid sequence analysis, functional domains prediction, and preparation of du-cGAS positive serum. **(A)** Multiple sequence alignments of cGAS sequences. Predicted domains are highlighted in different colors: NTase core (blue), Mab21 (red) and Zinc-Ribbon (green). Conserved residues are shaded in red. Conserved NLS sequence was highlighted by black frame. **(B)** A phylogenetic tree of the cGAS sequences. Black circle (•): the cloned duck cGAS. **(C)** Western blotting analysis of the mouse anti-Mab21 positive serum binding the endogenous du-cGAS protein.

The complete coding sequence of du-cGAS contains 1296 nucleotides, encoding a protein with 432 aa with a molecular weight of about 48.9 kDa (**Figures 1A, C**). Moreover, as shown in **Figure 1A**, du-cGAS could be divided into the N-terminus domain (NTase Core, 69aa-238aa) and C-terminus domain (Mab21, 121aa-401aa), with the latter also containing a Zinc-Ribbon domain (Zinc, 289aa-301aa). The CDS sequence of du-cGAS was submitted in the GenBank database (Submission ID: 2645032). There was 77.83% and 93.35% amino acid identity between the du-cGAS protein and the chicken (GenBank ID: XP_040522657) and goose (GenBank ID: XP_040409319) cGAS proteins, respectively. According to the phylogenetic analysis, cGAS homologs could be divided into four groups, including Birds, Primates, Rodentia, and Artiodactyla, whereby du-cGAS clustered in the Birds branch (**Figure 1B**).

### Generation of cGAS-KO DEF cell line

**Figure 2:**
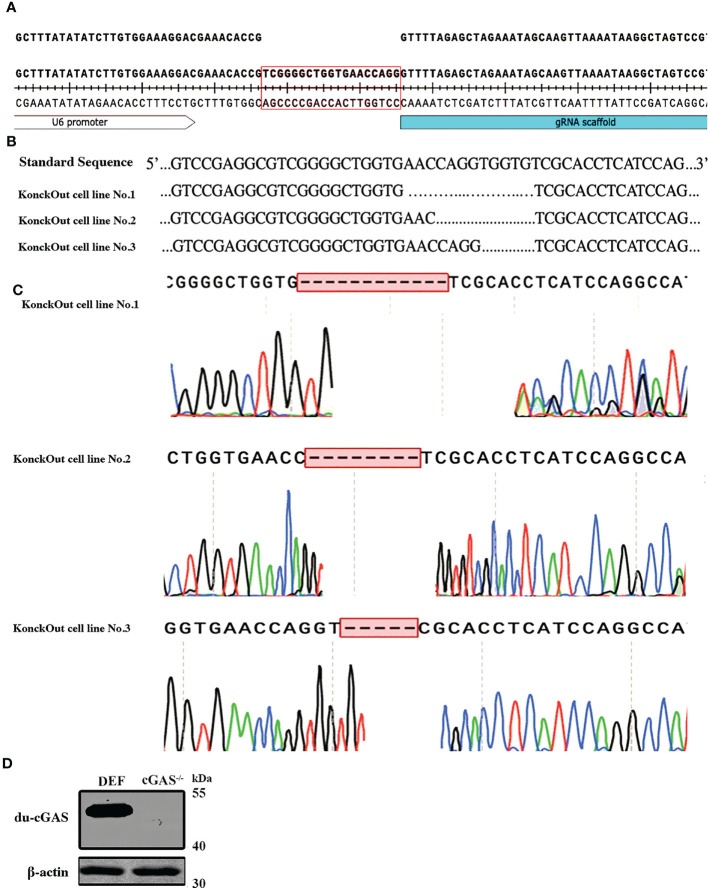
Generation and sequencing identification of the du-cGAS knockout DEF cells. **(A)** PAM sequence construction. (**B**, **C**) Genome sequencing of the knockout cell lines. **(D)** Western blotting analysis of the knockout efficiency.

To generate cGAS knockout in DEF cells, we utilized CRISPR-Cas9 gene editing technology to disrupt the translation of du-cGAS (**Figure 2A**). Thereafter, antibiotic-Puromycin (0.5ug/μL) incubation and subcloning were conducted to generate a stable cGAS-deficient DEF cell line. The DNA sequencing confirmed the presence of 5-12 bp deletion in cGAS deficient cell lines, which caused a frameshift mutation (**Figures 2B, C**). Additionally, no cGAS protein was detected in the cGAS deficient cell line, compared with wild-type DEF cells, *via* Western blot analysis (**Figure 2D**). These data showed a successful generation of the cGAS knockout (cGAS-KO) DEF cell line by the CRISPR/Cas9 system.

### Silencing the endogenous cGAS greatly promotes the replication of DNA viruses

**Figure 3:**
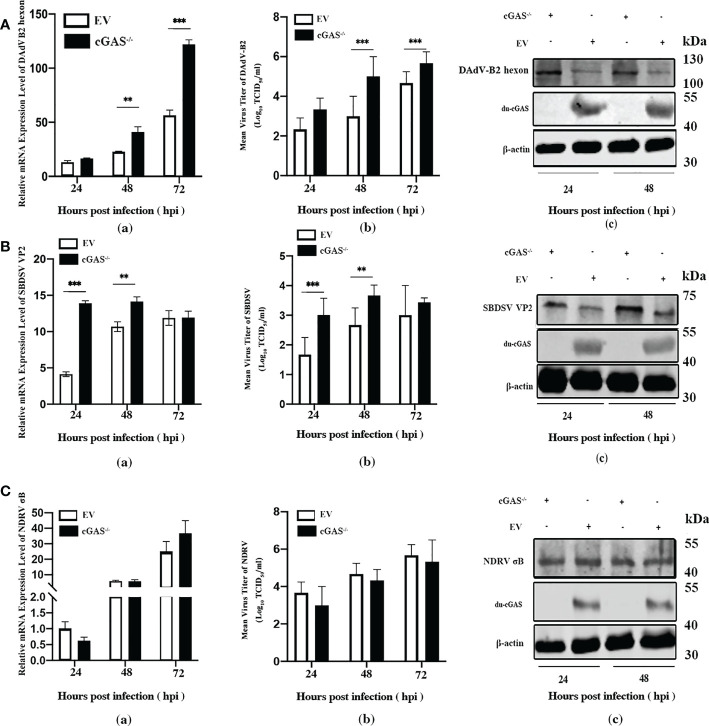
Knockout endogenous du-cGAS greatly promotes DNA viruses’ replication. The du-cGAS^-/-^ and EV DEF cells were infected with DAdV B2, SBDSV, and NDRV at the MOI of 0.25 and harvested at 24, 48, and 72 hpi respectively. The relative mRNA expression levels of the viral gene in the infected DEF cells were determined by qRT-PCR, and the viral titers in the cell culture supernatant were detected by TCID_50_ assay. The expression levels of viral protein in infected DEF cells were detected by western blotting. **(A)** DAdV-B2 infection. **(B)** SBDSV infection. **(C)** NDRV infection. Data represent the mean values ± SD, and statistical significance was analyzed by t-test. *P< 0.05, **P < 0.01, ***P < 0.001.

To explore the function of cGAS in response to viral infections, we infected the cGAS-KO DEF cells (cGAS^-/-^) and the normal control DEFs (cells transfected with empty vector pX459-v2, EV) with DAdV B2, SBDSV and NDRV viral strains at the MOI of 0.25 and harvested at the indicated time points. As shown in **Figure 3A-a**, the mRNA expression of the DAdV B2 hexon gene was significantly higher at 48 and 72 hpi compared to DEF-infected WT cells. The DAdV-B2-infected cGAS^-/-^ DEF cells had higher viral titers in cell culture supernatant than those of EV cells at 48 and 72 hpi (**Figure 3A-b**). Consistently, the expression of DAdV-B2 hexon protein was significantly increased in the cGAS^-/-^ DEF cells (**Figure 3A-c**). Consistently, silencing of the endogenous cGAS also increased the SBDSV mRNA expression and viral titer at 24 hpi, and significantly increased the SBDSV VP2 protein expression, as shown by qRT-PCR, TCID50, and Western blot analyses (**Figure 3B-a-c**). However, no significant differences were detected in NDRV sigma-B gene expression and viral titer between cGAS^-/-^ and EV DEF cells in response to NDRV infection (**Figure 3C-a-c**). These data suggest that cGAS deficiency could strongly promote the replication of duck-origin DNA viruses rather than dsRNA viruses.

### The expression of IFNs and ISGs was impaired in cGAS knockout DEF infected with the DNA viruses

**Figure 4:**
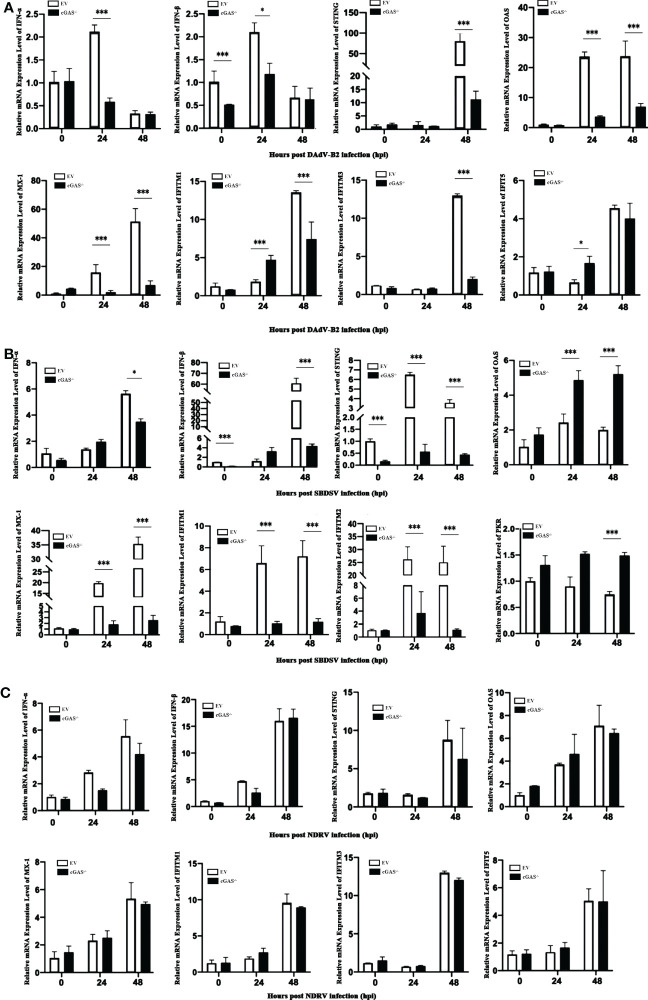
The expression levels of IFNs and ISGs in du-cGAS knockout DEFs after virus infection. Du-cGAS^-/-^ and EV DEFs were infected with DAdV-B2, SBDSV, and NDRV at the MOI of 0.25 and harvested at 24 and 48 hpi respectively. The relative mRNA expression levels of type I IFNs, STING and key ISGs in virus-infected DEFs were analyzed *via* qRT-PCR. **(A)** DAdV-B2 infection. **(B)** SBDSV infection. **(C)** NDRV infection. Data represent the mean values ± SD, and statistical significance was analyzed by t-test. *P < 0.05, **P < 0.01, ***P < 0.001.

Since cGAS deficiency greatly promotes the replication of DNA viruses rather than dsRNA viruses, we sought to explore the molecular mechanism underlying this replication enhancement. We evaluated the relative mRNA expression level of the vital genes associated with the cGAS-dependent signaling pathway *via* qRT-PCR analysis. As shown in **Figure 4A**, the mRNA expression levels of IFN-α and IFN-β were significantly lower in cGAS^-/-^ DEF cells than in EV DEF cells at 24 hpi in response to DAdV-B2 infection. Moreover, the mRNA expression of STING was significantly reduced in DAdV-B2 infected cGAS^-/-^ DEF cells at 48 hpi. The mRNA expression of vital ISGs, including Mx1, OAS, and IFITM1, were also greatly impaired in DAdV-B2 infected cGAS^-/-^ DEF cells at 24 and 48 hpi. Similarly, the mRNA expressions of STING, IFN-β, and ISGs (Mx1, OAS, IFITM1, and IFITM2) were highly inhibited in SBDSV-infected cGAS^-/-^ DEF cells at 24 and 48 hpi (**Figure 4B**). However, NDRV infection had no significant effect on the expression of IFNs (IFN-α and IFN-β), STING and the key ISGs (Mx1, OAS, IFITM1, IFITM2, IFITM3, and IFIT5) (**Figure 4C**). These data suggest that the increased viral replication in cGAS^-/-^ DEF cells was due to the destruction of cGAS/STING-dependent pathways, which resulted in the impaired expression of IFNs and ISGs.

### Du-cGAS overexpression strongly inhibited viral replication by upregulating the expression of IFNs and ISGs

**Figure 5:**
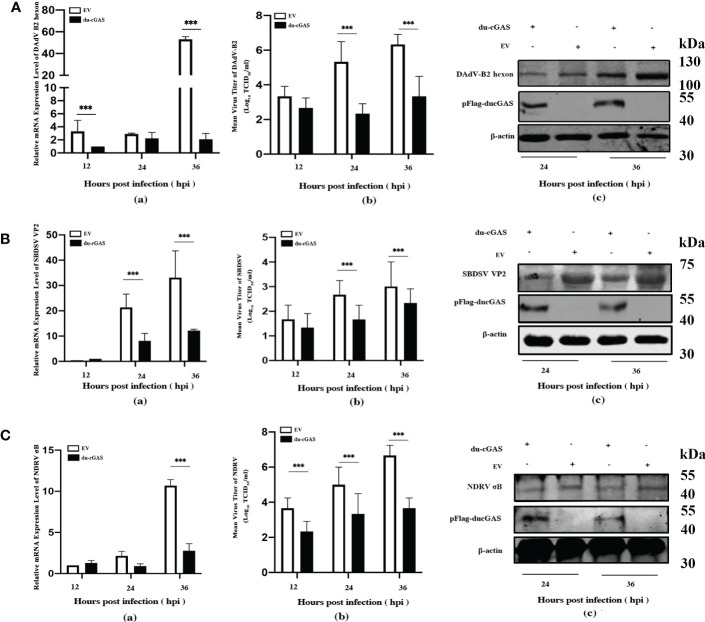
Du-cGAS overexpression inhibited both DNA and RNA viruses’ replication. DEF cells expressing du-cGAS and EV were infected with DAdV-B2, SBDSV and NDRV at the MOI of 1.0 and harvested at indicated time points (12, 24, and 36 hpi). The relative mRNA expression levels of viral genes in the infected DEF cells were determined by qRT-PCR, and the viral titers in the cell culture supernatant were detected by TCID_50_ assay. The viral protein expression levels in infected DEF cells were detected by western blotting. **(A)** DAdV-B2 infection. **(B)** SBDSV infection. **(C)** NDRV infection. Data represent the mean values ± SD, and statistical significance was analyzed by t-test. *P < 0.05, **P < 0.01, ***P < 0.001.

To further investigate the antiviral function of du-cGAS, we overexpressed the du-cGAS in DEF cells and infected the cells with DNA and RNA viruses. As shown in **Figure 5A-a**, viral copy numbers of DAdV B2 significantly decreased at 12 and 36 hpi compared to control DEFs cells (cells transfected with empty vector pFlag-CMV5a, EV). Overexpressing du-cGAS also suppressed DAdV-B2 replication, lowering the TCID_50_ titers in culture supernatants (**Figure 5A-b**). Consistently, du-cGAS overexpression in the DEFs significantly reduced DAdV-B2 replication, decreasing the expression of hexon protein, as determined by western blotting analysis (**Figure 5A-c**). SBDSV replication was also inhibited by du-cGAS overexpression, significantly lowering its viral mRNA expression level and viral titer (**Figure 5B-a, b**). Consistent with the results of TCID_50_ assay and qRT-PCR, the western blotting analysis showed that the expression of SBDSV VP2 protein was decreased in du-cGAS-overexpression cells compared to EV cells (**Figure 5B-c**). Overexpressing du-cGAS also significantly inhibited NDRV replication, as determined by qRT-PCR, TCID_50_ assay, and western blotting results (**Figure 5C-a, b, c**).

**Figure 6:**
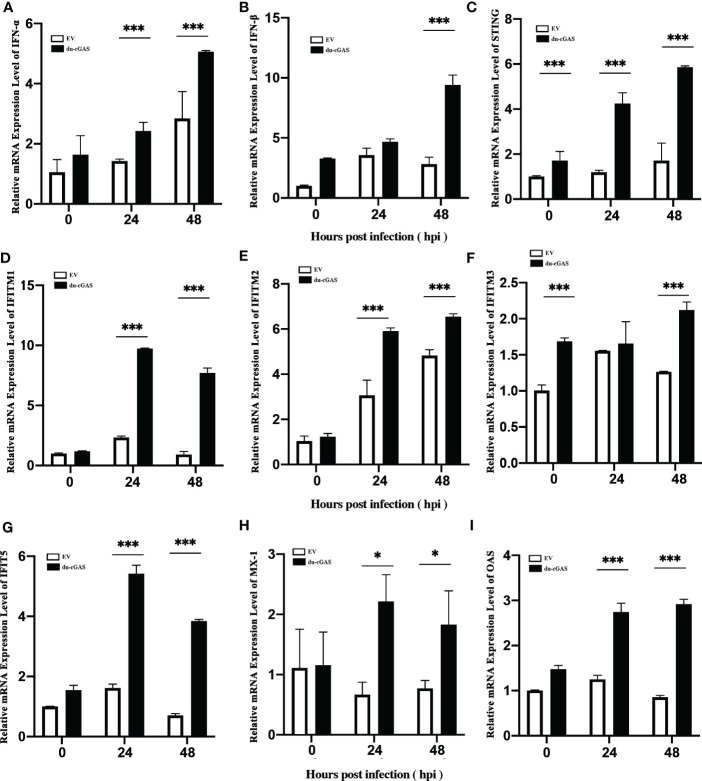
Activation of IFNs, STING, and key ISGs expression by du-cGAS overexpression. DEF cells expressing du-cGAS and EV were infected with DAdV B2 at the MOI of 1.0 and harvested at indicated time points (24 and 36 hpi). The qRT-PCR was used to detect the relative mRNA expression levels of type I IFNs, STING, and key ISGs. **(A)** IFN-α; **(B)** IFN-β **(C)** STING; **(D)** IFITM1; **(E)** IFITM2; **(F)** IFTIM3; **(G)** IFIT5; **(H)** Mx1; **(I)** OAS. Data represent the mean values ± SD, and statistical significance was analyzed by t-test. *P < 0.05, **P < 0.01, ***P < 0.001.

To clarify the possible antiviral mechanism of du-cGAS, qRT-PCR was used to detect the the relative mRNA expression levels of IFNs, STING, and key ISGs in DAdV-B2 infected cGAS-overexpression and EV DEFs. As expected, the mRNA expression levels of IFN-α, IFN-β, STING, OAS, Mx1, IFITM1, IFITM2, IFITM3, and IFIT5 were greatly enhanced in cGAS-overexpression DEFs than those in EV DEFs at 24 and 48 hpi (**Figures 6A–I**). These results indicate that du-cGAS is a vital antiviral component of the innate immune system of ducks.

### The NTase domain and Mab21 domain cooperate to inhibit virus replication

**Figure 7:**
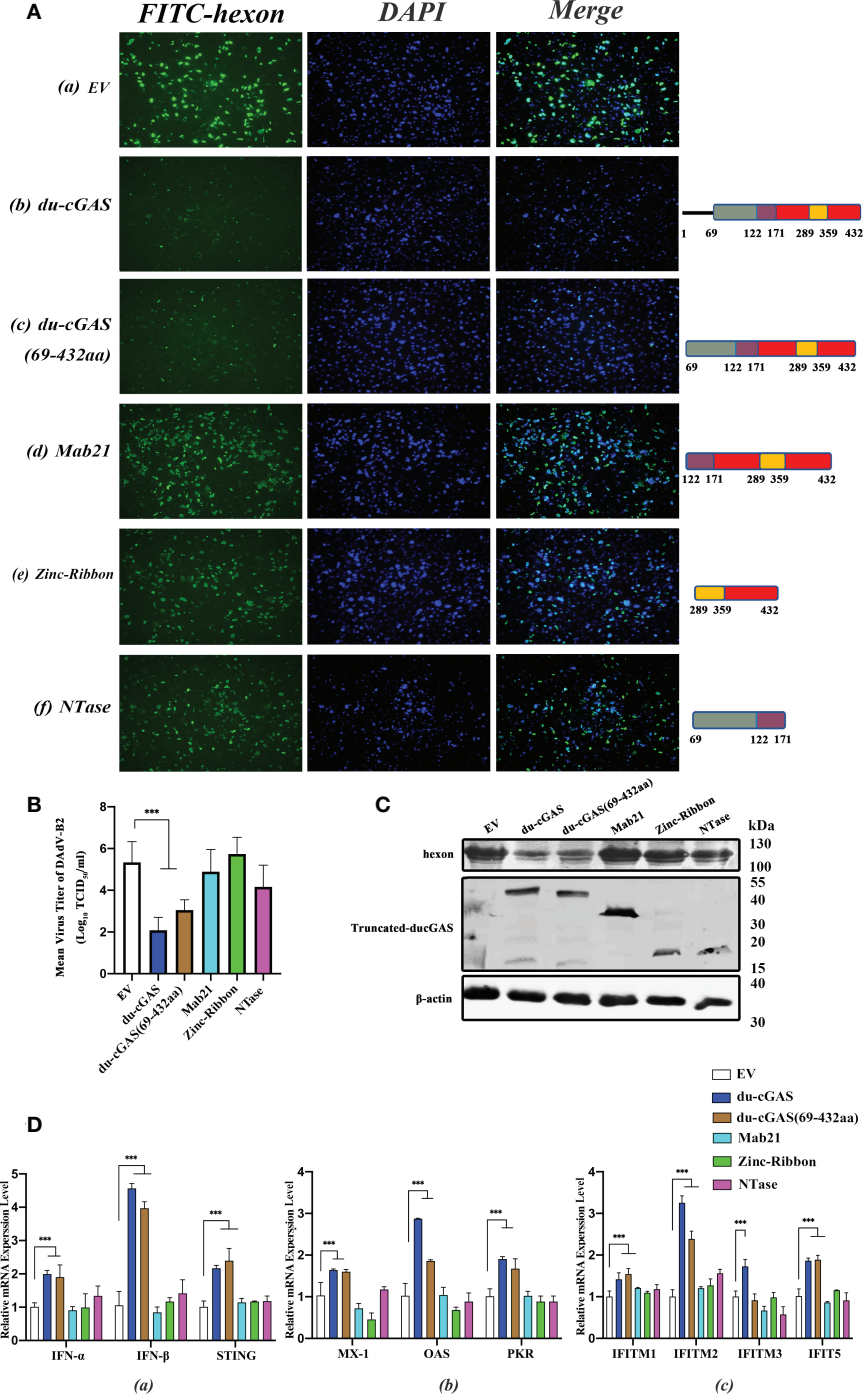
Characterization of du-cGAS functional domains. DEFs cells were transfected with expression plasmids encoding full-length du-cGAS, truncated du-cGAS or empty vector (EV). After 24 h post-transfection, cells were infected with DAdV-B2 at the MOI of 1.0 and harvested at 48 hpi. **(A)** Cell monolayers were detected the expression of DAdV-B2 hexon protein by IFA. **(a)** empty plasmid; **(b)** du-cGAS; **(c)** du-cGAS (69-423aa) **(d)** Mab21; **(e)** Zinc-Ribbon; **(f)** NTase. **(B)** The viral titers in the cell culture supernatant were detected by TCID_50_ assay. **(C)** The protein expression of full-length and truncated du-cGAS, and DAdV-B2 hexon was detected by western blotting. **(D)** The relative mRNA expression of type I IFNs, STING and key ISGs was detected by qRT-PCR. Data represent the mean values ± SD, and statistical significance was analyzed by t-test. *P < 0.05, ** *P* < 0.01, ***P < 0.001.

Since the preceding findings have shown that du-cGAS plays an important antiviral role in viral infections, we determined the effects of the NTase domain and Mab21 domains of du-cGAS on virus replication by constructing eukaryotic expression plasmids of truncated du-cGAS proteins. As shown in **Figure 7A b, c**, induced expression of full-length du-cGAS and du-cGAS (69aa-423aa) could strongly inhibit the expression of DAdV-B2 hexon protein as detected by IFA. However, induced sole expression of NTase, Mab21 and Zinc-Ribbon proteins of du-cGAS had no significant effects on the the expression of DAdV-B2 hexon protein. In order to further confirm this result, the viral loads were detected by TCID_50_ assay in the cell culture supernatant and the expression of DAdV-B2 hexon was also detected by western blotting. Consistently, forced expression of full-length du-cGAS and du-cGAS (69aa-423aa) could significantly reduce the viral loads with lower TCID_50_ titers, as compared to those in NTase, Mab21, Zinc-Ribbon overexpression and EV DEFs (**Figure 7B**). Similarly, the expression of DAdV-B2 hexon protein was obviously reduced in full-length du-cGAS and du-cGAS (69aa-423aa) overexpression DEFs as detected by western blotting (**Figure 7C**). To further address the correlation of these truncated du-cGAS proteins with the antiviral function, qRT-PCR was performed to detect the relative mRNA expression of IFNs, STING and key ISGs in DEFs transfected with these truncated du-cGAS plasmids. As shown in **Figure 7D**, the relative mRNA expression levels of IFNs-α, STING and key ISGs were greatly induced in du-cGAS and du-cGAS (69aa-423aa) overexpression DEFs. No significant up-regulation was observed in NTase, Mab21, Zinc-Ribbon overexpression DEFs. These results indicate that a relatively intact cGAS domain including NTase and Mab21 domains are required for the antiviral function of du-cGAS.

## Discussion

cGAS acts as a PRR and binds pathogenic DNA to trigger an innate immune response through the production of cGAMP, which activates the adaptor STING. The cGAS-STING pathway is involved in the type I interferon and ISGs responses against microbial infections such as chicken adenovirus 4 and Marek’s Disease Virus in fibroblasts (7, 20). In addition to sensing microbial DNA, this pathway is also involved in the defense against RNA virus infections (28, 29). Although the functions of mammalian and chicken cGAS have been extensively studied, the function of du-cGAS in response to duck-origin virus infection remains unclear. In this study, du-cGAS was cloned and characterized. The recombinant Mab21 protein of du-cGAS was expressed in pET28a(+) as an antigen for the preparation of mouse hyperimmune serum. We found that the mouse anti-Mab21 sera could successfully recognize the endogenic cGAS protein.

To evaluate the antiviral function of du-cGAS, we detected the viral replication levels in du-cGAS knockout or overexpression DEFs in response to three duck-origin viruses (SBDSV, DAdV-B2 and NDRV). Indeed, knocking out the endogenous du-cGAS greatly enhanced the infection of the DNA viruses but had no significant effect on NDRV replication. Interestingly, the ectopic expression of du-cGAS greatly inhibited both DNA and RNA viruses’ replication. The results show that duck cGAS has a broad-spectrum antiviral activity, especially against DNA viruses.

The antiviral activity of cGAS is dependent on the activation of STING and the downstream signaling responses (10). In the present study, we systematically examined the mRNA expression profile of type I IFNs, STING, and downstream ISGs in du-cGAS^-/-^ or overexpression DEFs infected with RNA and DNA viruses. The STING, type I IFNs, and vital ISGs mRNA expression levels were significantly decreased in du-cGAS^-/-^ DEFs in response to DNA virus infection. However, there were no significant differences in the mRNA expression of STING, type I IFNs, and vital ISGs between NDRV-infected du-cGAS^-/-^ DEFs and EV control DEF cells. Thus, these results explain why du-cGAS knockout cells promoted DNA virus replication but not RNA virus. Furthermore, du-cGAS overexpression induced robust expression of type I IFNs, STING, and ISGs in DEFs, resulting in a wide range of antiviral activity against viruses, including RNA viruses. These results support the conclusion that cGAS inhibits RNA virus replication by upregulating STING and the downstream cytokines.

Three functional regions were predicted in the du-cGAS protein, including NTase Core, Mab21, and Zinc-Ribbon. Only induced expression of the full-length du-cGAS and du-cGAS (69aa-423aa) could efficiently inhibit DAdV-B replication. Sole overexpression of NTase, Mab21 or Zinc-Ribbon did not impair DAdV-B2 replication. These findings showed that a deletion of at least 1-68 aa in the N terminus could not impair du-cGAS antiviral function. These results also indicate that a relatively intact du-cGAS domain, including the coordination between NTase and Mab21 domains, is necessary for its antiviral activity.

cGAS has been described as a cytosolic DNA sensor and mainly localizes in the cytoplasm (30). Many recent studies revealed that cGAS is localized in both cytoplasm and nucleus (31, 32). It has been proved that N-terminal residues (1-160 amino acids) of human cGAS play an important role in nuclear localization of cGAS and subsequent activation of STING/IRF3-mediated cytoplasmic DNA signaling (30, 31, 33). cGAS contains two nuclear localization sequences (NLS). In this study, two NLS motifs are also found in du-cGAS, ^7^RRQRAVRSKGSAGRGSSGGGEPREREGGPAGSGRGRPARAAPGGGRRPG^55^ (NLS1) and ^205^EVTVKRRKARS^315^ (NLS2). Interestingly, du-cGAS (69aa-423aa) deprived of the NLS1 motif had antiviral properties similar to those of full-length du-cGAS. Although we didn’t detect the nucleus location of du-cGAS (69aa-423aa) in this study, it is suggested that the the highly conserved NLS2 motif of du-cGAS may play a crucial role in nuclear localizing activities. Basing the low amino acid identity (48.98%) between duck and human cGAS, especially at N-terminal, du-cGAS may have evolved a unique regulatory modality that is different from mammalian cGAS. The specific mechanism of du-cGAS regulating antiviral innate immunity needs further study.

In summary, this is the first study to explore the role of du-cGAS in response to viral infections. Du-cGAS was cloned and characterized, and the du-cGAS knockout DEF cell line was generated using the CRISPR-Cas9 technique. Furthermore, the eukaryotic expression plasmids were constructed for expressing the full-length du-cGAS and truncated du-cGAS proteins. By analyzing the viral replication levels in du-cGAS knockout and overexpressed cell lines, it was found that du-cGAS is an important component of the innate immune system of ducks, with a universal antiviral activity. Importantly, this antiviral activity depends on the cGAS/STING-dependent pathway to activate the expression of IFNs and ISGs. These findings provide a useful strategy for effectively controlling waterfowl viral diseases.

## Data availability statement

The original contributions presented in the study are included in the article/supplementary material. Further inquiries can be directed to the corresponding authors.

## Ethics statement

The animal study was reviewed and approved by the Ethics Committee of the Institute of Animal Husbandry and Veterinary Medicine, Fujian Academy of Agriculture Sciences.

## Author contributions

SYC and SLC conceived and designed the study. SLC drafted the manuscript. CL and MZ performed most of the experiments and analyzed the data. SX, DJ, SW, XLZ and XC participated in the experiments. XCZ conceived the study and critically revised the manuscript. All authors contributed to the article and approved the submitted version.
